# Impact of COVID-19 Related Work Model Changes on Service Pathways for New Patient Assessments at East Kent Neuropsychiatry Service: Service Evaluation Comparing One Year Pre-COVID-19 Lockdown With One Year Post-Lockdown

**DOI:** 10.1192/bjo.2022.411

**Published:** 2022-06-20

**Authors:** Oluwaseun Olaluwoye, Verity Williams, Alan Dunlop, Rafey Faruqui

**Affiliations:** 1 Kent and Medway Partnership Trust, Kent, United Kingdom; 2 Kent and Medway Partnership Trust, Kent, USA; 3 Centre for Health Service Studies, University of Kent, Kent, United Kingdom; 4 Kent and Medway Medical School, Kent, United Kingdom

## Abstract

**Aims:**

Neuropsychiatry Service in East Kent typically receives referrals for patients with brain injury, progressive neurological conditions, epilepsy specific neuropsychiatric conditions, rare forms of dementia, and functional neurological conditions. COVID-19 pandemic disrupted routine functioning of the service requiring multiple service innovations including introduction of remote access assessments, skills development clinics, and video-conferencing based psychoeducation groups. We conducted a service evaluation with governance approval to understand the impact of COVID-19 work model changes on referral sources, patient attendance, discharge destinations and the mental health professionals’ involvement in the management of the patients referred to the service.

**Methods:**

We applied to Service Evaluation and Audit Group of Kent and Medway NHS Partnership Trust for permission to collect service data using routinely collected clinical and business administration information. We used an approved data collection form for anonymized data collection. We analysed data for new patient assessments conducted over one-year prior to COVID-19 lockdown announced on 23rd March 2020 and compared it with one-year post-COVID lockdown period ending on 22 March 2021. We used Statistical Package for Social Sciences (SPSS) to carry out descriptive and statistical analysis of the data from two service evaluation period.

**Results:**

The total number of new patient assessments conducted during the two designated service evaluation periods was 365. 233 new patient assessments (64%) were conducted during the one-year pre-COVID-19 lockdown and 132 (36%) new patient assessments were conducted during the one-year post-COVID-19 lockdown.

Neurology teams in the local area were the main source of referrals during the two study periods, referring 59% and 51% of total referrals during the two evaluation periods respectively. Other referral sources included local memory service, inpatient psychiatric units, community mental health teams, neuropsychology, neurorehabilitation, palliative care and acute medicine. The primary management model was multidisciplinary. 49% of assessment contacts were made by specialist nursing during the first evaluation period. 48% of assessment contacts were made by the medical staff during the post-lockdown period. 13.3% of patients did not attend their appointments during the first period, dropping to 9.8% in the Post-Lockdown period.

Most patients who completed treatment were discharged to GP care (89% pre-COVID-19 and 94% post-lockdown). 12% patients from Pre-Lockdown period were still receiving care at the end of one year and 35% were still receiving care in at the end of post-lockdown period.

**Conclusion:**

The service evaluation identifies systemic differences in service use characteristics during Pre-lockdown and Post-lockdown periods.

